# Tuberculosis among children and adolescents with rheumatic diseases - case series

**DOI:** 10.1186/s12969-023-00918-4

**Published:** 2023-11-10

**Authors:** Lenita de Melo Lima, Adriana Rodrigues Fonseca, Clemax Couto Sant’Anna, Ana Alice Amaral Ibiapina Parente, Rafaela Baroni Aurilio, Maria de Fátima Bazhuni Pombo Sant’Anna

**Affiliations:** 1 Pediatric Pulmonology Unit, Instituto de Puericultura e Pediatria Martagão Gesteira, Universidade Federal do Rio de Janeiro (UFRJ), Rua Bruno Lobo, 50 – Cidade Universitária, Rio de Janeiro, Brazil; 2 Pediatric Rheumatology Unit, Instituto de Puericultura e Pediatria Martagão Gesteira, Universidade Federal do Rio de Janeiro (UFRJ), Rua Bruno Lobo, 50 - Cidade Universitária, Rio de Janeiro, Brazil

**Keywords:** Mycobacterium tuberculosis, Pediatric rheumatic Diseases, Juvenile idiopathic arthritis

## Abstract

Background: Rheumatic patients have a higher frequency of tuberculosis(TB) than the general population. This study aimed to describe children and adolescents with TB and rheumatic diseases(RD) who were being treated in a reference center. Methods: A series of TB cases were investigated in a reference center for childhood TB in Rio de Janeiro, Brazil, from 1995 to 2022. Results: Fifteen patients with underlying RD and TB were included with 8(53%) being female. The mean age at RD diagnosis was 7.10years (SD ± 0,57 years), and the mean age at TB diagnosis was 9.81 years(SD ± 0.88 years). A total of 9 cases of pulmonary TB(PTB) and 6 cases of extrapulmonary TB—pleural(2), joint/osteoarticular(1), cutaneous(1), ocular(1), and peritoneal(1)- were described. The RD observed in the 15 patients included juvenile idiopathic arthritis(9), juvenile systemic lupus erythematosus(3), juvenile dermatomyositis(1), polyarteritis nodosa(1), and pyoderma gangrenosum(1). Among the immunosuppressants/immunobiologics, methotrexate(8) was the most commonly used, followed by corticosteroids(6), etanercept(2), mycophenolate mofetil(1), cyclosporine A(1), adalimumab(1), and tocilizumab(1). The most common symptoms were fever and weight loss, and a predominance of PTB cases was noted. GeneXpert MTB/RIF® was performed in six patients and was detectable in two without rifampicin resistance; Xpert Ultra® was performed in five patients, and traces with indeterminate rifampicin resistance were detected in three. One female patient discontinued treatment, and another passed away. Conclusions: The case series demonstrated the importance of suspecting and investigating TB in RD affected patients who are using immunosuppressants/ immunobiologics, particularly in countries with high rates of TB such as Brazil.

**Supplementary Information:**

The online version contains supplementary material available at 10.1186/s12969-023-00918-4.

## Background

Tuberculosis(TB) remains one of the top 10 causes of death in the world [1]. In Brazil, of the 78,057 new cases of TB diagnosed in 2022, 3.5%(2,703) occurred in children < 15 years of age [2].

Childhood TB is paucibacillary and collecting respiratory specimens properly is a challenge. In Brazil, the diagnosis of pulmonary TB(PTB) relies on the scoring system of the Brazilian Ministry of Health(MoH-Brazil) which does not require bacterial confirmation (Table 1) [3]. However, extrapulmonary TB(EPTB) may include microbiological tests and histopathological examination in this diagnosis [4].

**Table 1:** Brazilian National Ministry of Health Scoring System for the diagnosis of pulmonary tuberculosis in smear-negative children and adolescents

**Clinical manifestations**	**Points**
Fever or symptoms such as cough, adynamia, expectoration, weight loss and sweating for more than 2 weeks	+ 15
No symptoms or symptoms for less than 2weeks	0
Respiratory infection that improved with antibiotics for common pathogens or without antibiotics	-10
**Chest radiography findings**	**Points**
Hilar lymphadenopathy or milliary pattern AND/OR	+ 15
Condensation or infiltration (with or without cavitation) for more than 2 weeks, remaining unchanged AND/OR Condensation or infiltration (with or without cavitation) for more than 2 weeks, worsening or not improving with antibiotics for common pathogens	
Condensation or infiltration of any kind for less than 2 weeks	+ 5
Normal	-5
**History of contact with adult tuberculosis**	**Points**
Close contact in the last 2 years	+ 10
Occasional or no contact	0
**Tuberculin skin test results**	**Points**
Induration > or = 10 mm	+ 10
Induration between 5 and 9 mm	+ 5
Induration < 5 mm	0
**Nutritional status**	**Points**
Severe malnutrition	+ 5
Normal nutrition status or no severe malnutrition	0

Result interpretation: highly likely diagnosis: score > 40 points; possible diagnosis: score between 30 and 35 points; and unlikely diagnosis: score < or = 25 points

Adapted from: Brasil, Ministério da Saúde. Secretaria de Vigilância em Saúde. Departamento de Vigilância das Doenças Transmissíveis. Manual de Recomendações para o Controle da Tuberculose no Brasil. Brasília. Ministério da Saúde. 2020 [4]

Rheumatic patients have a higher frequency of TB than the general population **—**2 to 10 times higher in adults**—**which may be due to immunosuppression related to the underlying disease as well as the use of immunosuppressive and immunobiological medications [5, 6].

With the development of immunobiological drugs, an increased risk of infections has been observed [7–10]. Therefore, before beginning treatment, patients should undergo infectious surveillance and be screened for infections, such as TB [11].

This study aimed to describe children and adolescents with TB and rheumatic diseases(RD) in a reference center in Rio de Janeiro, Brazil.

## Methods

A series of TB patients aged 0 to 18 years were investigated in a reference center for childhood TB in Rio de Janeiro, Brazil, from 1995 to 2022. The pediatric rheumatology unit follows up children with several diseases with musculoskeletal manifestations. During 2022, 1992 patients were under observation: 431 juvenile idiopathic arthritis(JIA), 369 rheumatic fever, 193 juvenile systemic lupus erythematosus(JSLE), 92 autoinflammatory diseases, 90 juvenile dermatomyositis(JDM), 84 juvenile scleroderma, 74 Kawasaki disease, 66 other vasculitis, 17 mixed connective tissue disease and overlap syndromes, and 576 with others diagnosis. The project was approved by the Research Ethics Committee(CAAE45099121.3.0000.5264).

The diagnosis of PTB was based on a possible/probable classification based on the clinical score of the MoH-Brazil associated or not with a positive bacilloscopy and/or positive culture for *Mycobacterium tuberculosis(M.tb)* and/or a detected molecular rapid test (TB-MRT:GeneXpert MTB/RIF® or Xpert Ultra®); a positive tuberculin skin test(TST) was considered positive if the result was ≥ 5 mm [3].

The diagnosis of EPTB was based on radiographic findings, suggestive clinical findings and/or microbiological confirmation and/or histopathological examination compatible with TB.

The demographic data included patient’s age at TB diagnosis, age at diagnosis of the underlying RD, gender, and city of residence. Data was collected on medications in use, clinical manifestations, laboratory tests, and imaging and laboratory exams. The outcomes considered were death, treatment abandonment, and completion of the TB treatment.

For the treatment of RD, the following was considered: corticosteroids(prednisone or prednisolone at a dose of 2 mg/kg/day for a period of 14 days or more, or methylprednisolone pulse therapy), methotrexate(MTX), azathioprine, mycophenolate mofetil, leflunomide, cyclophosphamide, and cyclosporine A, and immunobiologics [tumor necrosis factor alpha blockers(anti-TNFα), interleukin6 inhibitors, co-stimulation inhibitors, interleukin1 inhibitors, and CD20 depleters].

The statistical analysis was descriptive, including mean and standard deviation(SD) (for normally distributed continuous variables), and absolute and percentage frequencies for categorical variables.

## Results

Fifteen patients with underlying RD and TB were included in the study with 8(53%) being female. The mean age of patients at RD diagnosis was 7.10 years(SD ± 0.57years), and the mean age at TB diagnosis was 9.81 years(SD ± 0.88years). A total of 9 cases of PTB and 6 cases of EPTB—pleural (2), joint/osteoarticular (1), cutaneous (1), ocular (1), and peritoneal (1) were included in the study.

The RD of these 15 patients included JIA [9], JSLE [3], JDM [1], polyarteritis nodosa [1], and pyoderma gangrenosum [1]. One patient was not taking immunosuppressive medication at the time of TB diagnosis and did not have a history of previous treatment for RD once the TB and RD were diagnosed simultaneously.

Among the immunosuppressants/immunobiologics, MTX [8] was the most commonly used, followed by corticosteroids [6], etanercept [2], mycophenolate mofetil [1], cyclosporine A [1], adalimumab [1], and tocilizumab [1]. The results are presented in Table 2 (Supplementary Material).

Of the 15 patients, 2 cases of TB occurred during JIA flare. One patient with pyoderma gangrenosum was reacting well to treatment. Twelve cases were in RD activity - according to disease activity instruments recommended for each disease). The results are presented in Table 2 (Supplementary Material).

Ten patients had symptoms suggestive of TB(7 PTB and 3 EPTB). Among the PTB, there were: fever [7], weight loss [6], cough [3], adynamia [1] and hemoptysis [1]; in EPTB cases, fever was the most common [3], followed by weight loss [1] and night sweating [1]. Three patients(3/6) with EPTB and two cases of PTB(2/9) had no TB symptoms. Among physician exam alterations, were observed: reduced or abolished lung auscultation [5], hepatomegaly/hepatosplenomegaly [4], tachypnea [3], hypoxia [3], ascites [3] and discomfort breathing [2]. TST was positive in 7/13. The scoring system of MoH-Brazil was possible or highly probable diagnosis in 7/8 of PTB cases.

Anemia was found in 5/15 patients − 1 JDM in use of MTX; 1 JSLE in use of corticosteroids and hydroxychloroquine; 3 JIA in use of: corticosteroid and cyclosporine, corticosteroid and MTX, and MTX. Low platelets were present in association with anemia in 1/15 patients (JSLE in use of corticosteroids and hydroxychloroquine); there were no cases of leukopenia or co-infection with human immunodeficiency virus or other opportunistic infections.

The bacilloscopy and culture for *M.tb* were negative in all 11 cases where they were performed. GeneXpert MTB/RIF® was performed in six patients and was detectable in two without rifampicin resistance; Xpert Ultra® was performed in five patients, and traces with indeterminate rifampicin resistance were detected in three.

One female patient discontinued treatment, and another passed away. The deceased was a 7-year-old patient with persistent polyarteritis nodosa activity (diagnosed in 2016) and was being treated with corticosteroids, MTX, and etanercept. In 2019, the patient developed miliary TB and macrophage activation syndrome(MAS).

## Discussion

The majority of patients in the present study had JIA and were using non-biological immunosuppressive medication. The most common symptoms were fever and weight loss, and a predominance of cases of PTB was noted.

The MoH-Brazil scoring system resulted in a possible or highly probable diagnosis of TB in 88% of PTB cases, highlighting the method’s significance in diagnosing PTB in children.

The TB-MRT was positive in 45% of the samples, which is a similar percentage to the EPTB cases in the previous study conducted in our institution [12]. However, our patients whose samples were sent for bacilloscopy and culture were negative, which reinforces the fact that children are paucibacillary [3].

Laboratorial alterations found could be explained by the RD itself, as well as the treatment involved. MTX and hydroxychloroquine are known for their hematological adverse effects as: anemia, leukopenia and low platelets [13, 14].

From the 1992 patients with RD followed-up at the pediatric rheumatology unit, 8 developed TB between 1995 and 2020 and 7 developed TB between 2021 and 2022, corresponding to 0,83% of the exposed patients. Our finding is similar to a study conducted in Portugal with adults under TNF-therapy from 2006 to 2019 [15].

In our cohort, we had a presumed prevalence of 33,3% (15/45) of TB disease. The higher prevalence of TB could be explained by the degree of immunosuppression in patients with RD and the medications they were taking [5, 6, 16] In addition, the study was conducted in a reference center for childhood TB, with a high number of the disease in this age group. As well, in childhood TB, approximately 20% of cases are EPTB [3]. However, in this study, 40% of patients with RD were diagnosed with EPTB.

In our case series, MTX was the most used medication for the treatment of RD, which could be explained by the period of the research (from 1995 to 2022), once the first biological agents, of the anti-TNF class, began to be prescribed in 2004, with still limited access, in this pediatric rheumatology unit. Furthermore, MTX is the first-line treatment prescribed for most cases of JIA [17]. Also, all patients were screened for latent TB and TB previous to any specific treatment for a given RD.

Despite screening prior to treatment, the use of anti-TNFα medications is associated with an increased risk of TB activation. Additionally, false negative TST due to immunosuppression caused by the RD or previous immunosuppressive therapy is a possibility [18].

In our case series, the fatal case of polyarteritis nodosa and miliary TB had factors that are known triggers of MAS. In an integrative review on TB in children with RD, the only reported death occurred in a patient who sequentially used etanercept, adalimumab, and abatacept and had EPTB [19]. Both patients underwent anti-TNFα therapy, which may be related to the severity of TB.

The limitations of our study include the small sample size and retrospective design, which presents challenges in obtaining some data due to incomplete information registration.

## Conclusion

The case series demonstrated the importance of suspecting and investigating TB in RD in patients who are using immunosuppressants/immunobiologics. The symptoms may be nonspecific; however, fever and weight loss should be taken seriously, leading to suspicion of TB disease. The scoring system of the MoH-Brazil is important for the diagnosis of PTB, while TB-MRT are increasingly playing a crucial role in the diagnosis of EPTB.

### Electronic supplementary material

Below is the link to the electronic supplementary material.

Supplementary Material 1

Supplementary Material 2

## Data Availability

All relevant data are reported in the article. Additional details can be provided by the corresponding author upon reasonable request.

## Abbreviations

Anti-TNFα: tumor necrosis factor alpha blockers
EPTB: extrapulmonary tuberculosis
JDM: juvenile dermatomyositis
JIA: juvenile idiopathic arthritis
JSLE: juvenile systemic lupus erythematosus
MAS: macrophage activation syndrome
MoH-Brazil: Brazilian Ministry of Healthy
M.tb: Mycobacterium tuberculosis
MTX: methotrexate
PTB: pulmonary tuberculosis
RD: rheumatic diseases
SD: standard deviation
TB: tuberculosis
TB-MRT: molecular rapid tests for tuberculosis
TST: tuberculin skin test
