# Supplementary material for: Tuberculosis among children and adolescents with rheumatic diseases - case series
Source: Pediatr Rheumatol Online J. 2023 Nov 10;21:136. doi: 10.1186/s12969-023-00918-4 (PMC10636992; doi:10.1186/s12969-023-00918-4)
Supplement: Supplementary file 2 — Supplementary Material 2 [file 12969_2023_918_MOESM2_ESM.docx]

**Tuberculosis among children and adolescents with rheumatic diseases - case series**

**Complete given names and surnames of all authors with ORCID ID:**

Lenita de Melo Lima¹-ORCID ID 0000-0001-5621-6204

Adriana Rodrigues Fonseca²-ORCID ID 0000-0002-3536-3552

Clemax Couto Sant’Anna¹-ORCID ID 0000-0001-8732-8065

Ana Alice Amaral Ibiapina Parente¹-ORCID ID 0000-0003-1349-7311

Rafaela Baroni Aurilio¹-ORCID ID 0000-0003-2727-520X

Maria de Fátima Bazhuni Pombo Sant’Anna¹-ORCID ID 0000-0002-3633-6070

1. Pediatric Pulmonology Unit, Instituto de Puericultura e Pediatria Martagão Gesteira, Universidade Federal do Rio de Janeiro (UFRJ). Rio de Janeiro – Brazil

Postal address: Rua Bruno Lobo, 50 – Cidade Universitária - Rio de Janeiro – Brazil Postal Code: 21941-912

2. Pediatric Rheumatology Unit, Instituto de Puericultura e Pediatria Martagão Gesteira, Universidade Federal do Rio de Janeiro (UFRJ). Rio de Janeiro – Brazil

Postal address: Rua Bruno Lobo, 50 – Cidade Universitária - Rio de Janeiro – Brazil

Postal Code: 21941-912

**Corresponding author:**

Lenita de Melo Lima

Postal address: Rua Bruno Lobo, 50 – Cidade Universitária - Rio de Janeiro – Brazil

Postal Code: 21941-912

Phone: 55-21-984065328 E-mail address: nitamelo.le@gmail.com

**Type of manuscript –** short report.

Dear editors,

I am writing to submit the article entitled, “Tuberculosis among children and adolescents with rheumatic diseases - case series” for consideration by Pediatric Rheumatology Journal Online. I can confirm that the attached article is an original piece of work and that I am not submitting it to other journals for consideration.

In this article, we describe a series of tuberculosis cases in children and adolescents with rheumatic diseases treated in a reference center in Rio de Janeiro, Brazil, from 1995 to 2022.

The findings confirmed the importance of suspecting and investigating tuberculosis in rheumatic patients who are using immunosuppressants or immunobiologics, particularly in countries with high rates of tuberculosis such as Brazil.

There is not any benefits from commercial sources for the work reported in the manuscript, or any other financial interests, which could create a potential conflict of interest with regard to the work.

No specific funding was received from any bodies in the public, commercial or not-for-profit sectors to carry out the work described in this article.

All procedures were performed in accordance with Brazilian local ethics regulations. Locally appointed ethics committee has approved the research protocol, under number CAAE45099121.3.0000.5264.

All authors made substantial contribution to conception and design, execution, or analysis and interpretation of data; drafting the article or revising it critically; and reading and approval of the final version and with the listing of the authors.

The authors agree to bear the applicable publication charges if their manuscript is accepted for publication.

Please send all correspondence regarding the publication of this article to nitamelo.le@gmail.com. Thank you for your time and consideration of the article.

Sincerely,

Mrs Lenita de Melo Lima.
